# Treating intrusions, promoting resilience: an overview of therapies for trauma-related psychological disorders

**DOI:** 10.3402/ejpt.v5.26520

**Published:** 2014-12-09

**Authors:** Ulrich Schnyder

**Affiliations:** Department of Psychiatry and Psychotherapy, Zurich University Hospital, Zurich, Switzerland

**Keywords:** evidence-based interventions, trauma-focused CBT, cognitive therapy, prolonged exposure, CPT, NET, EMDR, BEPP, resilience, mini-interventions, trauma, PTSD

## Abstract

The efficacy of psychotherapeutic approaches in the treatment of posttraumatic stress disorder (PTSD) can be regarded as empirically demonstrated. Overall, effect sizes appear to be higher for psychotherapy than for medication. Many well-controlled trials with a mixed variety of trauma survivors have demonstrated that trauma-focused cognitive-behavioral therapy (TF-CBT) is effective in treating PTSD. Prolonged exposure therapy (PE) is currently seen as the treatment with the strongest evidence for its efficacy. Cognitive therapy (CT) and cognitive processing therapy (CPT), with their stronger emphasis on cognitive techniques, and Eye Movement Desensitization and Reprocessing (EMDR) seem equally effective. More recent developments include brief eclectic psychotherapy for PTSD (BEPP) and narrative exposure therapy (NET). Emerging evidence shows that TF-CBT can successfully be applied in PTSD patients suffering from severe comorbidities such as borderline personality disorder or substance abuse disorder (Schnyder & Cloitre, 2015). There is also a trend towards developing “mini-interventions,” that is, short modules tailored to approach specific problems. Moreover, evidence-based approaches should be complemented by interventions that aim at promoting human resilience to stress. Finally, given the globalization of our societies (Schnyder, 2013), culture-sensitive psychotherapists should try to understand the cultural components of a patient's illness and help-seeking behaviors, as well as their expectations with regard to treatment.
